# Changes of volatile substance composition during processing of nine-processed tangerine peel (Jiuzhi Chenpi) determined by gas chromatography-ion mobility spectrometry

**DOI:** 10.3389/fnut.2022.963655

**Published:** 2022-08-24

**Authors:** Manqin Fu, Yuehan Wang, Yuanshan Yu, Jing Wen, Meng Sam Cheong, Wai San Cheang, Jijun Wu

**Affiliations:** ^1^Sericultural & Agri-Food Research Institute, Guangdong Academy of Agricultural Sciences/Key Laboratory of Functional Foods, Ministry of Agriculture and Rural Affairs/Guangdong Key Laboratory of Agricultural Products Processing, Guangzhou, China; ^2^State Key Laboratory of Quality Research in Chinese Medicine, Institute of Chinese Medical Sciences, University of Macau, Taipa, Macao SAR, China

**Keywords:** nine-processed tangerine peel, terpenes, volatile components, GC-IMS, food processing

## Abstract

Nine-processed tangerine peel (Jiuzhi Chenpi in Chinese) is a famous Chinese traditional snack. The composition and contents of volatile substances during its processing is unclear. Gas chromatography combined with ion mobility spectrometry (GC-IMS) was applied to determine the characteristic changes of volatile components throughout the production process. Four stages such as untreated dry tangerine peel (raw material), debittered tangerine peel, pickled tangerine peel, and final product were examined. A total of 110 flavor compounds including terpenes, alcohols, aldehydes, ketones, esters, acids, and two others were successfully detected in tangerine peel samples across the various production stages. There were abundant amounts of terpenes contributing to the flavor, including limonene, gamma-terpinene, alpha-pinene, myrcene, beta-pinene, and alpha-thujene which were reduced at the later stage of production. Large amounts of esters and alcohols such as methyl acetate, furfuryl acetate, ethyl acetate, benzyl propionate, 2-hexanol, linalool, and isopulegol, were diminished at the early stage of processing, i.e., soaking for debittering. One the other hand, the final product contained increased amount of aldehydes and ketones including pentanal, hexanal, 2-hexenal, 2-heptenal (E), 2-pentenal (E), 1-penten-3-one, 6-methyl-5-hepten-2-one, 2-methyl-2-propenal, and 2-cyclohexen-1-one, and very high level of acetic acid. Present findings help to understand the formation of the unique flavor of nine-processed tangerine peel and provide a scientific basis for the optimization of processing methods and quality control.

## Introduction

Nine-processed tangerine peel (Jiuzhi Chenpi in Chinese) is a famous traditional snack in China, originated from the Chaoshan region in Guangdong Province in China. The term “nine-process” describes the complicated and rigorous production process, which involves picking up (CP1), soaking (CP2), keeping fresh, peeling, pickling (CP3), draining, seasoning, repeated drying, and lastly storage (aging; CP4) of tangerine peels (1). With the addition of licorice, salt, and sugar though the manufacturing produces, this preserved food shows a sweet, salty, aromatic, and pungent flavor.

Nine-processed tangerine peel has become a famous snack in China due to its characteristic flavor. However, there have been limited studies on its volatile constituents, which possess potent efficacy and affect the flavor of nine-processed tangerine peel. In particular, the impact of different manufacturing procedures on the contents of volatile components and thereby the flavor of final product remains unclear. Flavor is not only an indicator for evaluating the food quality, but also a key factor affecting consumer satisfaction and market value of food products. The volatile components vary due to different processing and storage methods. Throughout the complicated series of manufacturing procedures, the volatile components will be changed and result in the unique flavor of nine-processed tangerine peel. Moreover, the volatile ingredients may possess pharmacological effects as suggested in previous studies (2, 3).

New technique having gas chromatography combined with ion mobility spectrometry (GC-IMS) allows the separation and identification of flavor molecules based on their migration under an electric field at specific pressures and temperatures (4). GC-IMS has low detection limits and high sensitivity without any pre-treatment requirement, making it easy to use and advantageous for accurate characterization and quantification of the volatile components in the sample. GC-IMS can be used to classify and analyze samples according to the intensity of the signal peaks of each component in the sample through its accompanying software function, and to create fingerprint profiles. Displaying the results in color images, GC-IMS allows us to detect the differences among samples visually. With these aforementioned advantages, GC-IMS has widely been used in food quality analysis and volatile compound identification (5, 6).

A recent study by gas chromatography–olfactometry-mass spectrometry (GC-O-MS) has identified the presence of 58 volatile components and their changes in contents during the pickling and storage of nine-processed tangerine peel, among which D-limonene was the major volatile component, up to 81.84% for different storage time periods (7). However, the composition and contents of volatile substances at each stages of the processing of nine-processed tangerine peel is still unclear and need to be further investigated. In this study, we used GC-IMS to explore in depth the changes of volatile components during the processing of nine-processed tangerine peel. Four stages in the processing were selected for examination: untreated dry tangerine peel (raw material; CP1), debittered tangerine peel (CP2), pickled tangerine peel (CP3), and final product (CP4), as they are the most crucial stages affecting the flavor. This study explored the characteristic changes of the volatile components throughout the process to understand the formation of the unique flavor of nine-processed tangerine peel and provide a scientific basis for the optimization of processing methods and quality control.

## Materials and methods

### Sample preparation

In this study, all samples were provided by Guangdong Jiabao Group Co., Ltd. (Guangdong, China). In brief, the production process included picking up (CP1), soaking (CP2), keeping fresh, peeling, pickling (CP3), draining, seasoning, repeated drying, and lastly storage (aging; CP4). Picking up was to select thick dried tangerine peel obtained from ripened tangerine as raw material (CP1). Soaking in water could debitter and soften the tangerine peel whilst removing impurities (CP2). Then the tangerine peel were cut into filamentous or blocky shape. Pickling involved soaking with 8–10% salt solution to further remove bitter substances by high osmotic pressure and to break the fibers in tangerine peel (CP3), followed by draining of water and remaining salt solution. Licorice, cyclamate, vanillin, citric acid, and others were added for seasoning. The tangerine peel was dried to ∼38% moisture content using 45–50°C heat pump. Importantly, the nine-processed tangerine peel were stored at room temperature for 0.5–1 year so that various flavor substances could be transformed and released to form the unique flavor of final products, which were packaged for sale after passing the necessary food quality and safety tests (CP4). The tangerine peel samples were divided into four groups based on different stages in the processing of nine-processed tangerine peel: untreated dry tangerine peel (CP1), deastringent and debittered tangerine peel (CP2); pickled tangerine peel (CP3), and final product (CP4). Three independent batches of samples were obtained from each of these four stages of processing. The samples were powdered with a pulverizer (Qingdao Juchuang Environmental Protection Group Co., Ltd. JC-FW-200) and passed through a 100-mesh sieve for further GC-IMS analysis.

### Gas chromatography combined with ion mobility spectrometry analysis

Gas chromatography combined with ion mobility spectrometry analysis of tangerine peel samples were performed by FlavourSpec^®^ (GAS Dortmund Co., Dortmund, German), equipped with a PAL3 autosampler system (CTC Analytics AG, Zwingen, Switzerland). Each sample powder (1 g) was put in a 20 mL vial for analysis. Headspace sampling was selected as the injection method. After heating at an oscillatory rate of 500 rpm at 60°C for 15 min, a 200-μL sample headspace was automatically injected by a heated syringe (85 °C) into the GC-IMS system. The FS-SE-54-CB-1 capillary column (15 m × 0.53 mm i.d. × 1.0 μm) from CS-Chromatographie Service GmbH (Langerwehe, Germany) was heated at 60°C. Purified nitrogen gas (99.999% purity) was used as the carrier. The carrier gas flow rate was programmed as follows: the flow rate was 2 mL/min for the first 2 min, the internal linearity was increased to 10 mL/min within 8 min, followed by 100 mL/min within 10 min, and then reached 150 mL/min within the last 10 min. The drift tube was 9.8 cm long and operated at a 500 V/cm constant voltage with a 150 mL/min nitrogen flow at 45°C.

### Data analysis

Gas chromatography combined with ion mobility spectrometry technology was equipped with VOCal software for data collection and analysis, as well as NIST database and IMS database for quantitative analysis of volatile substances. The GC-IMS instrument also provided with three plugins: (a) Reporter plug-in for direct comparison among samples from 3D spectrum, 2D top view spectrum and differential map; (b) Gallery Plot plugin for fingerprint comparison; and (c) Dynamic principal component analysis (PCA) plugin for clustering samples into different types.

## Results and discussion

### Gas chromatography combined with ion mobility spectrometry topographic plots in different process of nine-processed tangerine peel

**FIGURE 1:**
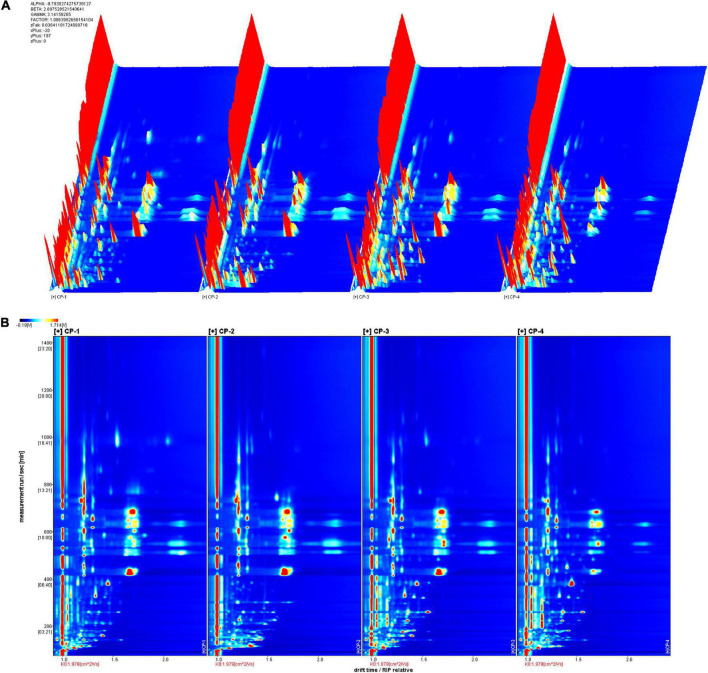
GC-IMS spectra of different tangerine peel samples. **(A)** 3D-topographic spectra. **(B)** 2D-topographic spectra. CP1, untreated dry tangerine peel raw material; CP2, deastringent and debittered tangerine peel; CP3, pickled tangerine peel; CP4, finished nine-processed tangerine peel.

**FIGURE 2:**
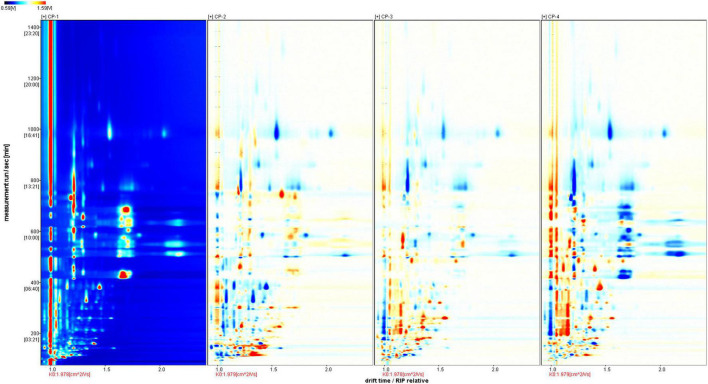
Differential spectra with CP1 as the reference for comparison results.

Four groups of samples represented different stages during the production of nine-processed tangerine peel. The changes of volatile substances were determined by GC-IMS analysis and clearly visualized in the 3D (Figure 1A) and 2D topographic spectra (Figure 1B). In the spectra, X-axis represents the drift time in the drift tube while Y-axis represents the retention time of gas chromatography. The red vertical line at abscissa 1.0 is reactive ion peak (RIP) after normalization of the data and blue color is set as background for the whole plot. Each volatile compound is present as a point on either side of RIP with color indication for its quantity, i.e., red indicates higher concentration, yellow and then white indicate lower concentration, and so on. The ionized molecules can be affected by factors including chemical properties, concentration of analyte, and temperature of drift tube (8).

To better identify the differences among the four samples, we used CP1 as a reference for topographic plot deduction to get a differential plot (Figure 2). In terms of signal intensity compared to the reference, blue means a lower concentration than the reference and the darker the color, the lower the concentration; and *vice versa*, red means a higher concentration than the reference and darker the color, the higher the concentration. For volatile compounds at the same concentration, the background is white after the deduction. In total, 110 volatile substances were identified in each tangerine peel sample from four different processing stages by identification approach based on the retention index (RI) and drift time in the drift tube (Dt), and were listed in Table 1. The substances were grouped by types with 18 of them unidentified. There were 9 terpenes, 12 alcohols, 17 aldehydes, 13 ketones, 8 esters, 2 acids, and 2 others detected. Dimers and trimers of some compounds were also detected.

**TABLE 1:** Quantitative analysis result of volatile compounds found in different processing stages of nine-processed tangerine peel using GC-IMS.

Count	Compound	CAS	Formula	MW	RI	Rt	Dt
**Terpenes**							
A1	α-Thujene	2867-05-2	C_10_H_16_	136.2	924.7	422.833	1.648
A2-1	α-Pinene M	80-56-8	C_10_H_16_	136.2	932.5	436.357	1.30375
A2-2	α-Pinene D	80-56-8	C_10_H_16_	136.2	932	435.47	1.66736
A2-3	α-Pinene T	80-56-8	C_10_H_16_	136.2	932.5	436.357	1.72396
A3-1	β-Pinene M	127-91-3	C_10_H_16_	136.2	978.5	517.051	1.30203
A3-2	β-Pinene D	127-91-3	C_10_H_16_	136.2	977.5	515.278	1.64334
A3-3	β-Pinene T	127-91-3	C_10_H_16_	136.2	975.2	511.287	2.17161
A4-1	Myrcene M	123-35-3	C_10_H_16_	136.2	997.4	550.304	1.29002
A4-2	Myrcene D	123-35-3	C_10_H_16_	136.2	997.8	551.191	1.71195
A4-3	Myrcene T	123-35-3	C_10_H_16_	136.2	997.8	551.191	2.15789
A5	α-Phellandrene	99-83-2	C_10_H_16_	136.2	1010.9	577.35	1.67079
A6-1	α-Terpinene M	99-86-5	C_10_H_16_	136.2	1025.3	606.17	1.2077
A6-2	α-Terpinene D	99-86-5	C_10_H_16_	136.2	1024.6	604.681	1.71794
A7-1	Limonene M	138-86-3	C_10_H_16_	136.2	1038.6	632.772	1.65192
A7-2	Limonene D	138-86-3	C_10_H_16_	136.2	1038.2	631.885	1.71881
A7-3	Limonene T	138-86-3	C_10_H_16_	136.2	1037.5	630.555	2.17332
A8-1	γ-Terpinene M	99-85-4	C_10_H_16_	136.2	1068.3	692.206	1.20715
A8-2	γ-Terpinene D	99-85-4	C_10_H_16_	136.2	1066.5	688.59	1.69814
A9-1	Terpinolene M	586-62-9	C_10_H_16_	136.2	1089.8	735.124	1.21366
A9-2	Terpinolene D	586-62-9	C_10_H_16_	136.2	1088.6	732.806	1.30326
**Alcohols**							
B1-1	Linalool M	78-70-6	C_10_H_18_O	154.3	1110.4	776.255	1.21548
B1-2	Linalool D	78-70-6	C_10_H_18_O	154.3	1107.3	770.118	1.74892
B2	Isopulegol	89-79-2	C_10_H_18_O	154.3	1152.8	861.158	1.3747
B3	Borneol	507-70-0	C_10_H_18_O	154.3	1175.3	906.231	1.21834
B4	α-Terpineol	98-55-5	C_10_H_18_O	154.3	1197.8	951.076	1.29654
B5	2-Butanol	78-92-2	C_4_H_10_O	74.1	559.7	117.653	1.32406
B6-1	2-methyl-1-propanol M	788-3-1	C_4_H_10_O	74.1	637.6	151.335	1.1681
B6-2	2-methyl-1-propanol D	78-83-1	C_4_H_10_O	74.1	635.4	150.383	1.36138
B7	1-Penten-3-ol	616-25-1	C_5_H_10_O	86.1	696.1	179.351	0.93973
B8	3-Methyl-2-butanol	598-75-4	C_5_H_12_O	88.1	621	144.163	1.4322
B9	2-Methyl butanol	137-32-6	C_5_H_12_O	88.1	742.9	216.088	1.23414
B10	*cis*-3-Hexenol	928-96-1	C_6_H_12_O	100.2	881.3	353.972	1.23288
B11-1	2-Hexanol M	626-93-7	C_6_H_14_O	102.2	831.8	300.22	1.28466
B11-2	2-Hexanol D	626-93-7	C_6_H_14_O	102.2	833	301.553	1.56872
B12	2-Butoxyethanol	111-76-2	C_6_H_14_O_2_	118.2	904.8	387.814	1.20594
**Aldehydes**							
C1-1	Decanal M	112-31-2	C_10_H_20_O	156.3	1215.4	986.349	1.5437
C1-2	Decanal D	112-31-2	C_10_H_20_O	156.3	1213.6	982.696	2.0479
C2	2-Methyl-2-propenal	78-85-3	C_4_H_6_O	70.1	582.1	127.314	1.22025
C3	2-Methylpropanal	78-84-2	C_4_H_8_O	72.1	576.3	124.825	1.27826
C4	Butanal	123-72-8	C_4_H_8_O	72.1	607.1	138.132	1.28476
C5	3-Methylbutanal	590-86-3	C_5_H_10_O	86.1	659.5	160.844	1.39437
C6-1	Pentanal M	110-62-3	C_5_H_10_O	86.1	701.2	183.32	1.18719
C6-2	Pentanal D	110-62-3	C_5_H_10_O	86.1	700.6	182.88	1.41874
C7-1	Furfural M	98-01-1	C_5_H_4_O_2_	96.1	863.4	334.487	1.08407
C7-2	Furfural D	98-01-1	C_5_H_4_O_2_	96.1	859.7	330.521	1.32875
C8	2-Pentenal (E)	1576-87-0	C_5_H_8_O	84.1	753.4	224.318	1.35919
C9	3-Methyl-2-butenal	107-86-8	C_5_H_8_O	84.1	786.8	251.299	1.35486
C10-1	2-Hexenal M	505-57-7	C_6_H_10_O	98.1	851.6	321.706	1.17969
C10-2	2-Hexenal D	505-57-7	C_6_H_10_O	98.1	850.8	320.825	1.51342
C11	2-Methylpentanal	123-15-9	C_6_H_12_O	100.2	756.3	226.627	1.52389
C12	Hexanal	66-25-1	C_6_H_12_O	100.2	796	261.328	1.5603
C13-1	2-Heptenal (E) M	18829-55-5	C_7_H_12_O	112.2	960.3	485.128	1.25572
C13-2	2-Heptenal (E) D	18829-55-5	C_7_H_12_O	112.2	960	484.685	1.66049
C14	Heptanal	111-71-7	C_7_H_14_O	114.2	903.7	386.051	1.34374
C15	p-methylbenzaldehyde	104-87-0	C_8_H_8_O	120.2	1096.2	747.904	1.59076
C16	(E, E)-2,4-Non-adienal	5910-87-2	C_9_H_14_O	138.2	1198.6	952.677	1.34405
C17	n-Non-anal	122-63-4	C_9_H_18_O	142.2	1107.5	770.473	1.48045
**Ketones**							
D1	2-Propanone	67-64-1	C_3_H_6_O	58.1	545.5	111.483	1.11407
D2	2,3-Butanedione	431-03-8	C_4_H_6_O_2_	86.1	597	133.78	1.17184
D3	2-Butanone	78-93-3	C_4_H_8_O	72.1	602.6	136.186	1.24077
D4-1	2-Pentanone M	107-87-9	C_5_H_10_O	86.1	693.3	177.15	1.11969
D4-2	2-Pentanone D	107-87-9	C_5_H_10_O	86.1	693.1	176.943	1.36822
D5-1	1-Penten-3-one M	1629-58-9	C_5_H_8_O	84.1	690.5	174.947	1.07751
D5-2	1-Penten-3-one D	1629-58-9	C_5_H_8_O	84.1	693.3	177.15	1.30906
D6	2,3-Pentadione	600-14-6	C_5_H_8_O_2_	100.1	657.8	160.082	1.30972
D7-1	Mesityl oxide M	141-79-7	C_6_H_10_O	98.1	798	263.531	1.11313
D7-2	Mesityl oxide D	141-79-7	C_6_H_10_O	98.1	796	261.328	1.4403
D8-1	Cyclohexanone M	108-94-1	C_6_H_10_O	98.1	900.7	380.763	1.15532
D8-2	Cyclohexanone D	108-94-1	C_6_H_10_O	98.1	900.7	380.763	1.44686
D9	Methyl isobutyl ketone	108-10-1	C_6_H_12_O	100.2	736.1	210.755	1.47472
D10-1	2-Cyclohexen-1-one M	930-68-7	C_6_H_8_O	96.1	942.2	453.363	1.11409
D10-2	2-Cyclohexen-1-one D	930-68-7	C_6_H_8_O	96.1	940	449.534	1.40417
D11	2-Heptanone	110-43-0	C_7_H_14_O	114.2	893.6	368.218	1.26133
D12	6-Methyl-5-hepten-2-one	110-93-0	C_8_H_14_O	126.2	997.2	549.991	1.17213
D13	Acetophenone	98-86-2	C_8_H_8_O	120.2	1088.6	732.714	1.18838
**Esters**							
E1	Benzyl propionate	122-63-4	C_10_H_12_O_2_	164.2	1302	1159.553	1.36339
E2	Methyl acetate	79-20-9	C_3_H_6_O_2_	74.1	559.9	117.711	1.19134
E3-1	Ethyl acetate M	141-78-6	C_4_H_8_O_2_	88.1	614	141.145	1.09629
E3-2	Ethyl acetate D	141-78-6	C_4_H_8_O_2_	88.1	613.1	140.727	1.3335
E4	Ethyl butanoate	105-54-4	C_6_H_12_O_2_	116.2	756.8	226.958	1.56823
E5-1	Methyl 2-furoate M	611-13-2	C_6_H_6_O_3_	126.1	970.4	502.863	1.14938
E5-2	Methyl 2-furoate D	611-13-2	C_6_H_6_O_3_	126.1	971.9	505.523	1.46497
E6	Diethyl malonate	105-53-3	C_7_H_12_O_4_	160.2	1057.1	669.678	1.24525
E7	Isobutyl propanoate	540-42-1	C_7_H_14_O_2_	130.2	834.1	302.677	1.70958
E8-1	Furfuryl acetate M	623-17-6	C_7_H_8_O_3_	140.1	1014.2	584.001	1.41351
E8-2	Furfuryl acetate D	623-17-6	C_7_H_8_O_3_	140.1	1015.5	586.661	1.81314
**Acids**							
F1-1	Acetic acid M	64-19-7	C_2_H_4_O_2_	60.1	732.1	207.56	1.04938
F1-2	Acetic acid D	64-19-7	C_2_H_4_O_2_	60.1	736	210.645	1.15344
F2-1	2-Methylbutyric acid M	116-53-0	C_5_H_10_O_2_	102.1	834.8	303.494	1.20754
F2-2	2-Methylbutyric acid D	116-53-0	C_5_H_10_O_2_	102.1	833	301.526	1.49239
**Others**							
G1	Propylsulfide	111-47-7	C_6_H_14_S	118.2	866.8	338.27	1.16162
G2	Benzothiazole	95-16-9	C_7_H_5_NS	135.2	1215.6	986.706	1.16348
**Unknowns**							
H1	1	Unidentified	*	0	557.9	116.846	1.26463
H2	2	Unidentified	*	0	575.5	124.49	1.1166
H3	3	Unidentified	*	0	620.5	143.955	0.9469
H4	4	Unidentified	*	0	618.6	143.135	1.20856
H5	5	Unidentified	*	0	616.2	142.087	1.39371
H6	6	Unidentified	*	0	616.7	142.315	1.58081
H7	7	Unidentified	*	0	731.1	206.822	1.20163
H8	8	Unidentified	*	0	738.4	212.567	1.39851
H9	9	Unidentified	*	0	759.7	229.26	1.22288
H10	10	Unidentified	*	0	773.7	240.288	1.34396
H11	11	Unidentified	*	0	854	324.351	1.42062
H12	12	Unidentified	*	0	887.1	360.253	1.20217
H13	13	Unidentified	*	0	903.1	384.925	1.5661
H14	14	Unidentified	*	0	1013.4	582.375	1.50821
H15	15	Unidentified	*	0	1077.2	709.988	1.29904
H16	16	Unidentified	*	0	1089.7	734.884	1.71586
H17	17	Unidentified	*	0	1260.6	1076.828	1.30874
H18	18	Unidentified	*	0	1269.2	1093.944	1.4464

Monomers, dimers, and trimers of compound formed in the IMS drift tube were represented by M, D, and T, respectively. MW, molecular weight; RI, retention index; Rt, retention time in the capillary column; Dt, drift time in the drift tube; unknown formula was represented by symbol “*”.

Ion mobility spectrometry is a fast and sensitive way to detect molecules with low concentration (ppbv levels) (9). Combining the separation properties of GC with the fast response of IMS, GC-IMS can effectively employed for identification and differentiation of volatile compounds in foods. A great variety in the volatile compositions was observed across the processing of nine-processed tangerine peel. Some detected compounds gradually increased while other flavor compounds diminished with the depth of processing; such changes were further illustrated in details in the following analyses. Previous studies have determined the volatile profiles consisting of 51 compounds in varieties of dried tangerine peels by gas chromatography-mass spectrometry (GC-MS) analysis (10, 11). The components were predominantly terpenes such as D-limonene, γ-terpinene, α-pinene, and β-pinene. In addition, a recent report have detected a total of 58 volatile components in nine-processed tangerine peel during pickling and storage by gas chromatography–olfactometry-mass spectrometry (GC-O-MS) (7). Present findings were in line with the reported results and particularly, more flavor compounds were identified. These results supported the advantages of GC-IMS for trace gas analysis compared with GC-MS or GC-O-MS. GC-MS and GC-O-MS have drawbacks, as they require pre-treatments of the samples, such as solvent extraction, supercritical fluid extraction, water vapor distillation and solid phase microextraction (SPME) (12). These complicated treatments may affect the reflection of the true gaseous composition in its natural state. Besides, these techniques require a long detection interval, which is not conducive to the rapid analysis of volatile substances. Therefore, GC-IMS has been increasing applied for examining the volatile profile in foods, as for example, extra virgin olive oils (13), and Dezhou braised chicken (14). Recently, we have examined the impact of different drying methods on the volatile flavor components in nine-processed tangerine peel by GC-IMS, revealing that those prepared by baking has a richer aroma than those prepared by sun-drying (15). Currently, we did not determine the effects of drying methods but the effects of soaking, pickling, and aging to compare with the raw material. Similar but more volatile flavor components were detected in this study. Of note, compounds labeled with number 1–18 were unidentified in the tangerine peel samples, which should be studied in the future.

### Composition of volatile substances in different stages of nine-processed tangerine peel processing

**FIGURE 3:**
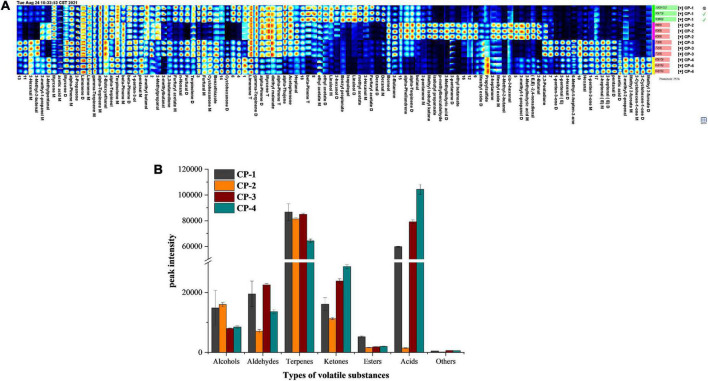
Changes of volatile substances during processing of nine-processed tangerine peel. **(A)** Fingerprint spectrum of volatile compounds in four groups of samples. **(B)** Summarized data showing peak intensities of different types of volatile substances in different process stages.

During the whole process of nine-processed tangerine peel production, flavor substances detected by GC-IMS consist of alcohols, aldehydes, carboxylic acids, esters, ketones, terpenes, and other compounds. The Gallery Plot analysis as fingerprinting technique was applied to provide overall and intuitive analysis of the composition changes in flavor substances during the processing (16). We selected the signal peaks of volatile substances in the spectrum of each sample to form the fingerprint spectrum (Figure 3A). Each row in the figure represents all signal peaks in one sample, and each column represents the same volatile substances in different samples. According to the whole spectrum analysis, the composition of volatile substances was different during the production stages of nine-processed tangerine peel. Furthermore, the peak intensities of volatile substances categorized by types for the four stages were summarized in Figure 3B.

Firstly, the concentrations of favor substances including limonene, gamma-terpinene, alpha-pinene, myrcene, beta-pinene, alpha-thujene, acetophenone, diethyl malonate, and heptanal marked in the red frame were the lowest in the final products. These substances are mainly terpenes, indicating that terpenes will lose during the processing of nine-processed tangerine peel and the loss mainly happened at the later stage of production. Terpenes are important aroma and flavor compounds with characteristic odors, flavors, and colors. In accordance with the previous reports, D-limonene, γ-terpinene, α-pinene, β-myrcene, β-pinene, and α-thujene were abundant in citrus peel (17, 18). Microorganims may play crucial roles in transformation of terpenes to other compounds (19).

A number of enzymatic reactions and metabolic pathways for transformation of terpenes have been identified. For instance, pinene with isomers α-pinene and β-pinene is the most abundant bicyclic monoterpene. In *Pseudomonas rhodesiae* and *P. fluorescens*, α-pinene can be oxidized to α-pinene oxide by a NADH-dependent α-pinene oxygenase and also form isonovalal or novalal by α-pinene oxide lyase (20). Alternatively, α-pinene can be transformed into limonene and pinocarveol but β-pinene can only form pinocarveol in *Bacillus pallidus*. Limonene is the most abundant monocyclic monoterpene, which transforms into limonene-1,2-diol through limonene-1,2-epoxide by limonene-1,2 monooxygenase and limonene-1,2-epoxide hydrolase in *Rhodococcus erythropolis* DCL14. Besides, limonene can be hydroxylated by a NADPH-dependent limonene 6-monooxygenase to *trans*-carveol, which is oxidized to carvone and dihydrocarvone by carveol dehydrogenase and carvone reductase respectively. The field of the microbial transformation of terpenes is not fully understood and remains to be explored.

Secondly, methyl acetate, furfuryl acetate, ethyl acetate, benzyl propionate, 2-hexanol, linalool, isopulegol, and decanal shown in the yellow frame mainly were present in the raw material CP1 samples. They are mostly esters and alcohols, suggesting that a large number of esters and alcohols in the tangerine peel will reduce rapidly at the early stage of processing.

Next, tangerine peel CP2 samples in the second processing stage after debittering contained a great number of volatile substances with dynamic composition. The contents of (E, E)-2,4-non-adienal, butanal, p-methylbenzaldehyde, isobutyl propanoate, ethyl butanoate, 2-butanol, borneol, 2-methyl-1-propanol, 3-methyl-2-butanol, *cis*-3-hexenol, 2-butanone, 2-pentanone, 2-heptanone, methyl isobutyl ketone, mesityl oxide, 2-methylbutyric acid, alpha-terpinene, and alpha-phellandrene marked in the green frame were more abundant in the second stage than other production stages. Among these flavor substances, p-methylbenzaldehyde, ethyl butanoate, mesityl oxide, propylsulfide, 2-heptanone, 3-methyl-2-butanol, *cis*-3-hexeno were only found in the samples of the second stage. On the other hand, substances in the purple frame consisting 2,3-butanedione, furfural, benzothiazole, n-non-anal, and terpinolene showed an opposite trend, being absent in the second stage.

Lastly, the contents of pentanal, hexanal, 2-hexenal, 2-heptenal (E), 2-pentenal (E), 1-penten-3-one, and 6-methyl-5-hepten-2-one detected were higher in samples of the last two production stages. Furthermore, volatile substances such as acetic acid, 2-methyl-2-propenal, methyl 2-furoate, 2-cyclohexen-1-one gradually increased and reached the highest concentration during the processing of nine-processed tangerine peel. Aldehydes may be formed from lipid oxidation and Maillard reactions, which are also known as non-enzymatic browning reactions in foods, resulting in browned food with characteristic flavor (21). Aldehydes are widely considered to be the critical contributors to the overall flavor among all categories due to the relatively low odor thresholds. For instance, hexanal can be derived from linoleic acid and exhibits a low odor threshold of 1.1 ng/L, affecting rice aroma (22).

To sum up, the final product of nine-processed tangerine peel contained reduced amount of terpenes, esters and alcohols, which were lost during processing; whereas the final product contained increased amount of aldehydes and very high level of acetic acid. The preserved food contained abundant terpenes and acids, which are particularly important contributors for the unique odors, flavors, and colors.

### Similarity analysis of volatile substances in different process stages

**FIGURE 4:**
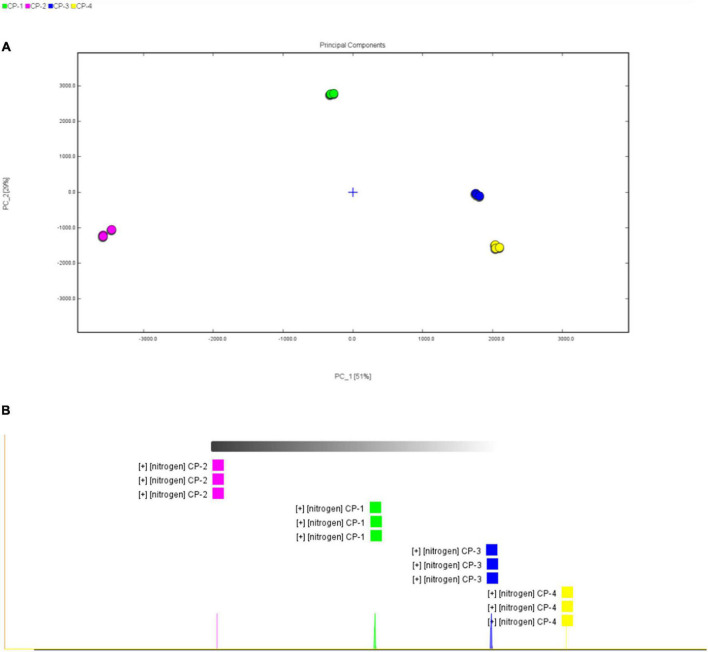
Similarity analysis of volatile substances in different process stages. **(A)** Score plot of principal component analysis (PCA) based on the GC-IMS signal intensity in samples from different process stages of nine-processed tangerine peel. **(B)** Euclidean distance analysis.

Principal component analysis (PCA) is a classification method that can effectively highlight the similarities or differences among datasets, commonly applied in chemometrics and bioinformatics (23, 24). Converting the original variables into clusters based on chemical properties, the PCA result of the volatile substances in different stages was shown in Figure 4A. All domains for four different stages were clearly separated among one another.

Euclidean distance analysis apart from PCA was applied further for similarity analysis and the results were shown in Figure 4B and Table 2. Euclidean distance is defined as the distance between two points or between the point and the origin (25). Euclidean distances of all samples were measured, indicating that the compositions of volatile substances between processing stages were significant different. The results of both PCA and Euclidean distances analysis indicated that there were apparent differences in the volatile substance profiles among four processing stages, and hence the processing stages can be distinguished according to the composition of volatile substances.

**TABLE 2:** Euclidean distances of all samples.

Full distance matrix												
	CP-1	CP-1	CP-1	CP-2	CP-2	CP-2	CP-3	CP-3	CP-3	CP-4	CP-4	CP-4
CP-1	0	119370	192178	14613582	15703209	15907904	10840446	11484577	11449610	12009563	12273046	12394705
CP-1	119370	0	29797	14689902	15746088	15945320	10613743	11137686	11123435	12140832	12455323	12605156
CP-1	192178	29797	0	14978902	16026481	16220378	10705903	11194838	11164050	12239860	12563456	12722572
CP-2	14613582	14689902	14978902	0	64884	87369	18269112	18804734	18737792	17628378	17808262	18138770
CP-2	15703209	15746088	16026481	64884	0	7907	19262936	19772207	19703642	18557243	18734433	19085370
CP-2	15907904	15945320	16220378	87369	7907	0	19439997	19942994	19873779	18672611	18848059	19203784
CP-3	10840446	10613743	10705903	18269112	19262936	19439997	0	127152	133663	6731959	7164820	7402765
CP-3	11484577	11137686	11194838	18804734	19772207	19942994	127152	0	18491	7220292	7680411	7987983
CP-3	11449610	11123435	11164050	18737792	19703642	19873779	133663	18491	0	7011537	7452277	7742947
CP-4	12009563	12140832	12239860	17628378	18557243	18672611	6731959	7220292	7011537	0	61667	105910
CP-4	12273046	12455323	12563456	17808262	18734433	18848059	7164820	7680411	7452277	61667	0	60230
CP-4	12394705	12605156	12722572	18138770	19085370	19203784	7402765	7987983	7742947	105910	60230	0

## Conclusion

The present study determined the complexity of volatile compounds at key processing stages throughout the manufacturing of nine-processed tangerine peel. A total of 110 flavor compounds were successfully detected by HG-GC-IMS in tangerine peel samples across the various production stages. The distinctive flavor of nine-processed tangerine peel is attributed to the unique set of volatile compounds. The limitations of the present study are that we did not perform quantitative analysis for the various volatile flavor components and did not determine the transformation pathways of the compounds happened during the manufacturing processes, which should be explored in the future.

## Data availability statement

The raw data supporting the conclusions of this article will be made available by the authors, without undue reservation.

## Author contributions

MF, YY, and JinW performed the experiments and analyzed the data. YW, MC, and WC prepared the manuscript. MF and JijW revised the manuscript. All authors read and approved the final manuscript.
